# CT-based peritumoral radiomics signatures to predict early recurrence in hepatocellular carcinoma after curative tumor resection or ablation

**DOI:** 10.1186/s40644-019-0197-5

**Published:** 2019-02-27

**Authors:** Quan-yuan Shan, Hang-tong Hu, Shi-ting Feng, Zhen-peng Peng, Shu-ling Chen, Qian Zhou, Xin Li, Xiao-yan Xie, Ming-de Lu, Wei Wang, Ming Kuang

**Affiliations:** 1 Department of Medical Ultrasonics, Institute of Diagnostic and Interventional Ultrasound, Ultrasomics Artificial Intelligence X-Lab, the First Affiliated Hospital of Sun Yat-Sen University, 58 Zhong Shan Road 2, Guangzhou, 510080 China; 2 Department of Radiology, the First Affiliated Hospital of Sun Yat-Sen University, 58 Zhong Shan Road 2, Guangzhou, 510080 China; 3 Clinical Trials Unit, the First Affiliated Hospital of Sun Yat-sen University, 58 Zhong Shan Road 2, Guangzhou, 510080 China; 4 GE Healthcare, Shanghai, China; 5 Department of Liver Surgery, Division of Interventional Ultrasound, the First Affiliated Hospital of Sun Yat-Sen University, 58 Zhong Shan Road 2, Guangzhou, 510080 China

**Keywords:** Hepatocellular carcinoma, Recurrence, Tomography, Radiomics

## Abstract

**Objective:**

To construct a prediction model based on peritumoral radiomics signatures from CT images and investigate its efficiency in predicting early recurrence (ER) of hepatocellular carcinoma (HCC) after curative treatment.

**Materials and methods:**

In total, 156 patients with primary HCC were randomly divided into the training cohort (109 patients) and the validation cohort (47 patients). From the pretreatment CT images, we extracted 3-phase two-dimensional images from the largest cross-sectional area of the tumor. A region of interest (ROI) was manually delineated around the lesion for tumoral radiomics (T-RO) feature extraction, and another ROI was outlined with an additional 2 cm peritumoral area for peritumoral radiomics (PT-RO) feature extraction. The least absolute shrinkage and selection operator (LASSO) logistic regression model was applied for feature selection and model construction. The T-RO and PT-RO models were constructed. In the validation cohort, the prediction efficiencies of the two models and peritumoral enhancement (PT-E) were evaluated qualitatively by receiver operating characteristic (ROC) curves, calibration curves and decision curves and quantitatively by area under the curve (AUC), the category-free net reclassification index (cfNRI) and integrated discrimination improvement values (IDI).

**Results:**

By comparing AUC values, the prediction accuracy in the validation cohort was good for the PT-RO model (0.80 vs. 0.79, *P* = 0.47) but poor for the T-RO model (0.82 vs. 0.62, *P* < 0.01), which was significantly overfitted. In the validation cohort, the ROC curves, calibration curves and decision curves indicated that the PT-RO model had better calibration efficiency and provided greater clinical benefits. CfNRI indicated that the PT-RO model correctly reclassified 47% of ER patients and 32% of non-ER patients compared to the T-RO model (P < 0.01); additionally, the PT-RO model correctly reclassified 24% of ER patients and 41% of non-ER patients compared to PT-E (*P* = 0.02). IDI indicated that the PT-RO model could improve prediction accuracy by 0.22 (P < 0.01) compared to the T-RO model and by 0.20 (*P* = 0.01) compared to PT-E.

**Conclusion:**

The CT-based PT-RO model can effectively predict the ER of HCC and is more efficient than the T-RO model and the conventional imaging feature PT-E.

**Electronic supplementary material:**

The online version of this article (10.1186/s40644-019-0197-5) contains supplementary material, which is available to authorized users.

## Introduction

Hepatocellular carcinoma (HCC) is the fifth most common type of cancer [1] and the third leading cause of cancer-related deaths in China [2]. When possible, resection and ablation are treatment options for primary HCC [1]. However, the prognosis of HCC remains poor due to the high frequency of early recurrence (ER) [3–6], which means that the recurrence after resection or ablation occurs within two years. MVI is a histopathological diagnosis based on peritumoral tissues, and as it is generally known that microvascular invasion (MVI) is the major risk factor affecting the ER of HCC [7–11], peritumoral tissues might have valuable predictive information of HCC prognosis. It is important to identify imaging biomarkers for predicting MVI preoperatively. Several studies have reported that certain imaging findings based on the peritumoral tissues, including peritumoral enhancement (PT-E) and peritumoral hypointensity (PT-H), in the hepatobiliary phase are useful for predicting MVI and ER in HCC [11–16]. However, the prediction accuracy of those conventional imaging features was not satisfactory, which may be attributed to the subjective or qualitative characteristics of conventional imaging features.

Radiomics is a new method for medical image analysis, defined as the high-throughput extraction of quantitative metric features that result in the conversion of images into mineable data and the subsequent analysis of these data for decision support [17, 18]. The peritumoral region captured by radiomic analysis may possess valuable predictive information of treatment response and outcomes in glioblastoma multiforme and breast cancer [19, 20]. Researchers found that peritumoral radiomics (PT-RO) might further improve survival prediction over intratumoral radiomics (T-RO) and some clinical parameters. Available studies that preoperatively predicted recurrence and survival in HCC were all based on T-RO [21, 22], but the generalizability of their findings awaits further investigation due to a lack of validation. Therefore, we intend to use a new radiomics method to identify peritumoral imaging biomarkers for predicting ER in HCC.

In this study, we explored the application of PT-RO for the first time for the noninvasive prediction of ER after HCC resection or ablation based on pretreatment computed tomography (CT), and we used an independent validation group to assess its prediction accuracy.

## Materials and methods

### Patients

**Fig. 1:**
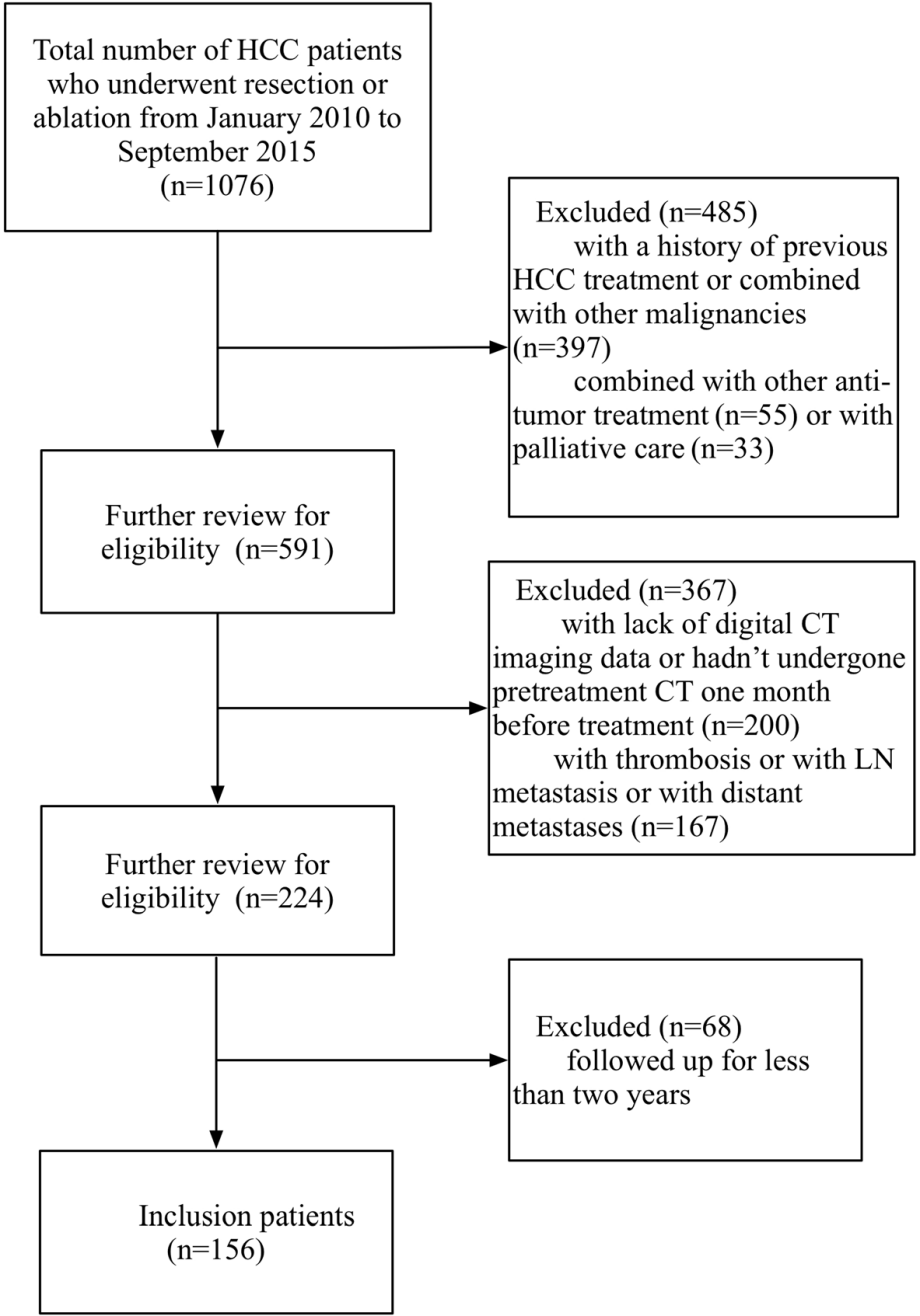
Flow diagram of the patient selection process. Abbreviations: *HCC* hepatocellular carcinoma, *CT* computed tomography, *LN* lymph node

This retrospective study was approved by our institutional review board and was conducted by searching for electronic medical records. A total of 1076 patients who underwent tumor resection or ablation at our institution with histopathologically confirmed HCC were recruited from January 2010 to September 2015. Figure 1 depicts the patient selection flow diagram. The inclusion criteria were as follows: (1) patients who had tumor resection or ablation with curative intent between January 2010 to September 2015 and (2) those who had preoperative CT performed within one month before treatment. Patients were excluded from the study if they met the following criteria: (1) those with a history of previous HCC treatment or a combination of other malignancies (*n* = 397); (2) those who received a combination of other anti-tumor treatments (*n* = 55), such as transarterial chemoembolization (TACE), targeting therapy, radiotherapy, and so on, or palliative care (*n* = 33); (3) patients who lacked digital CT imaging data or patients who did not undergo pretreatment CT 1 month before tumor resection or ablation (*n* = 200); (4) those with major thrombosis in a branch of the portal vein, hepatic vein thrombosis, or abdominal lymph node metastasis or distant metastases that were confirmed with pathology or imaging (*n* = 167); or (5) those who were followed up for less than 2 years (*n* = 68). Therefore, the final study population included 156 patients. The entire cohort was randomly divided into a training dataset (109 cases) and validation dataset (47 cases) by a ratio of 7:3. The training dataset was used to compose models that were evaluated by the validation dataset.

### Follow-up surveillance after tumor resection or ablation

Our post-treatment tumor surveillance program consisted of physical examinations and laboratory tests, including tests for serum alpha-fetoprotein (AFP), performed 1 month after surgery and then every 3 months thereafter. In addition, abdominal CECT, CEMR or CEUS imaging was performed every 3 months. The endpoint was ER, which was defined as the presence of new intrahepatic lesions or metastasis with typical imaging features of HCC, or atypical findings with histopathological confirmation within 2 years after curative resection or ablation of HCC.

### CT scan protocols

CECT was performed at our institute with one of the following machines: a 64-detector row (Aquilion CXL, Toshiba Medical System, Tokyo, Japan) or 320-detector row CT machine (Aquilion One, Toshiba Medical System, Tokyo, Japan). We used the same scanning parameters for both machines as follows: tube voltage, 120 kV; tube current, 250 mA; and slice thickness, 1 mm. After a routine unenhanced scan, 1.5 mL/kg of contrast media (Ultravist, Bayer, Germany) was injected into an antecubital vein at a rate of 3.0 mL/s via a pump injector (P3T abdomen module, Medrad Inc.). Hepatic arterial phase CT images were obtained at 35 s, and portal venous phase CT images were obtained at 65 s [23, 24].

### Image analysis

Two radiologists (S.T.F. and P.Z.P.), both with 15 years of abdominal CT interpretation, and both blinded to the clinical data, independently evaluated the imaging features randomly. The radiologists independently recorded incidences of PT-E (defined as detectable arterial-enhancing portions adjacent to the tumor border on arterial-phase images that became isodense with the background liver parenchyma on delayed-phase images [25]); when there were disagreements, they reached a consensus by discussion.

**Fig. 2:**
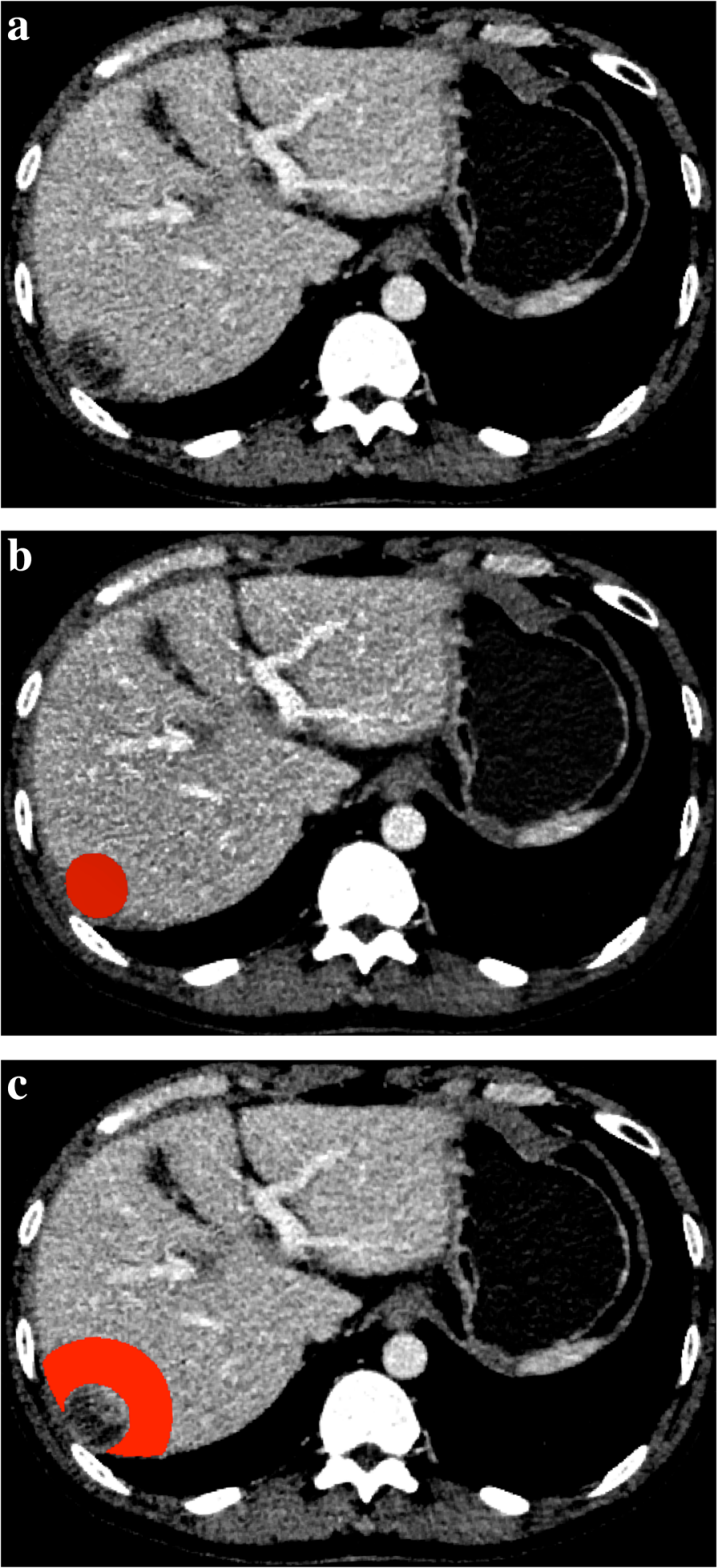
Drawing of the region of interest (ROI). A 65-year-old male with histopathologically confirmed hepatocellular carcinoma within segment 6/7 and a tumor size of 7.4 cm × 7.0 cm. (**a**) CT image (1 mm) of the largest cross-sectional area of the tumor in the routine unenhanced phase. (**b**) The manually delineated ROI around the lesion for the T-RO model. (**c**) The ROI for the PT-RO model was automatically expanded 2 cm from the lesion, and if the ROI was beyond the parenchyma of the liver after expansion, the portion beyond the parenchyma was manually removed

CT images (1 mm) on the largest cross-sectional area of the tumor, including routine unenhanced (Fig. 2a), hepatic arterial and portal venous phases, were recorded as digital imaging data and communications in medicine (DICOM) files. The slice chosen for delineating the lesion was confirmed by two radiologists in consensus.

### Radiomics features extraction and radiomics models building in the training set

DICOM images were used to extract radiomics features using A. K. software (Artificial Intelligence Kit, Version 1.0.0, GE Life Science, Institute of Precision Medicine), including routine unenhanced, hepatic arterial and portal venous phases. A T-RO region of interest (ROI) was manually delineated around the lesion (Fig. 2b). A PT-RO ROI of automatically expanded 2 cm from the lesion, and if the ROI was beyond the parenchyma of the liver after the expansion, the portion beyond the parenchyma was removed manually (Fig. 2c). The radiologists tried to keep ROIs in the three phases as consistent as possible.

Radiomics features were extracted from the ROIs using the A.K. software. A total of 1044 features were extracted from one single ROI, including four types of features: gray-level histogram texture, wavelet-transformed texture, transformed matrix texture, and filter-transformed texture. With the histogram texture, we extracted the texture feature parameters and made a quantitative or qualitative description of the texture based on the gray value of the images. With the wavelet-transformed texture, we analyzed the characteristics of the ROI through different levels of resolution. The transformed matrix texture reflected the high-level information of the ROI by a series of matrix transformations. With the filter-transformed texture, we obtained a series of target features by different types of filters.

Fifty patients were randomly selected, and their ROIs (containing T-RO and PT-RO) in the selected DICOM images were delineated by two radiologists (S.T.F. and P.Z.P.) blinded to the clinical data. Then, radiologist S.T.F. finished the final 106 patient ROIs. Radiomics features were automatically extracted from the ROIs by A. K. software through computing algorithms and recorded as comma separated values (CSVs).

The radiomics features extracted from the 50 patients by radiologist S.T.F. were compared with the features extracted by radiologist P.Z.P. using an independent sample t-test or a Kruskal-Wallis H test. Interclass correlation coefficients (ICCs) were used to assess the interobserver agreement of the feature extractions. Radiomics features with an ICC greater than 0.6 (indicating moderate-excellent agreement) were recorded for further analysis.

The linear regression least absolute shrinkage and selection operator (LASSO) regression was performed to select the features [26, 27] after manually eliminating the features that had an absolute value less than 0.6 for the coefficients of ER from the radiomics features extracted by radiologist S.T.F. in the training set of 109 patients. Finally, the PT-RO model was built using the selected features extracted from the ROIs of PT-RO, and the T-RO model was built using the selected features extracted from the ROIs of T-RO.

### Performance of the PT-RO model, T-RO model and PT-E

The PT-RO model, T-RO model and PT-E were first assessed in the training set and then validated in the independent validation set. The receiver operating characteristic (ROC) curve was plotted to show the prediction accuracy of predicting ER. Prediction accuracy was quantified with area under the curve (AUC). The more the ROC curve deviated from the baseline, the greater the AUC value was, which indicated higher accuracy of the prediction. The significant difference in AUC between the training and validation cohorts indicated overfitting. Calibrations (i.e., the agreement between observed outcome frequencies and predicted probabilities) were plotted to explore the predictive accuracy of the models in the validation cohort. The unreliability (U) statistic was used to assess the calibration, and *P* values of more than 0.05 were considered well-calibrated [28]. Decision curve analysis (DCA) was conducted to determine the clinical usefulness of the prediction models by quantifying the net benefits at different threshold probabilities in the validation cohort [29]. The more the curve deviated from the baseline, the greater the benefit was. The improvement in the predictive accuracy of the models was evaluated by calculating the integrated discrimination improvement (IDI) and the category-free net reclassification index (cfNRI). CfNRI generalizes to any upward or downward movement in predicted risks. IDI is the absolute value of the change in predicting accuracy.

### Statistical analysis

The baseline information in the training and validation cohorts were compared using the chi-squared test or the Fisher exact test for categorical variables and the two-sample t-test or the Mann–Whitney U test for continuous variables. *P* values of less than 0.05 (two-sided) were considered statistically significant. Computer-generated random numbers were used to assign 7/10 of the patients to the training dataset and 3/10 of the patients to the validation dataset. To test the intraobserver variability of the enhancement patterns, the intraclass correlation coefficient (ICC) was calculated. An ICC greater than 0.6 indicated moderate-excellent agreement.

The ROC curves were plotted to demonstrate the performance of the PT-RO model, T-RO model and PT-E in predicting ER in the training cohort and validation cohort, and AUC was used to evaluate the accuracy of the two models and PT-E in predicting the ER. Calibration curves were plotted to explore the predictive accuracy. DCA was conducted to determine the clinical usefulness by quantifying the net benefits at different threshold probabilities in the validation cohort. The improvement in the predictive accuracy of the models was evaluated by calculating IDI and cfNRI. CfNRI generalizes to any upward or downward movement in predicted risks. IDI is the absolute value of the change in predicting accuracy. The detailed methods introducing the calibration curves, DCA, cfNRI and IDI are provided in the Additional file 1.

All statistical analyses were conducted with the open-source statistical computing environment R (R Foundation for Statistical Computing, version 3.4.1; https://www.r-project.org/). The ICC was applied with the R package “irr”. Data cleaning was conducted using the R packages “knnImputation” and “DMwR”. The “glmnet” package of R was used for the LASSO regression. Univariate and multivariate logistic regressions were calculated and plotted using the R package “glm”. The “pROC” package was used to plot the ROC curves and measure the AUC. The “CalibrationCurves” package was used for the calibration curves. The “DecisionCurve” package was used to perform DCA. CfNRI and IDI were conducted with the R package “nricens” and “PredictABEL”.

## Results

### Patient characteristics

The baseline clinical information in the training and validation cohorts is summarized in Table 1. There was no significant difference between the training and validation cohorts for age (*P* = 0.29), gender (*P* = 0.25), AFP (*P* = 1.00), lesion diameter (*P* = 0.57), number of nodules (*P* = 0.35), treatment method (*P* = 0.15) and ER rate (*P* = 0.51). In addition, there were no significant differences between the two cohorts in positive PT-E rate, T-RO risk score and PT-RO risk score.

**Table 1:** Patient characteristics

Characteristics	Training Set (*N* = 109)	Validation Set (*N* = 47)	*P*
Gender (Male/ Female)	59/50	29/18	0.25
Age (Mean ± SD)	53.2 ± 12.4	55.4 ± 10.6	0.29
Preoperative AFP (Mean ± SD) (ng/mL)	946.7 ± 50,371.4	7891.4 ± 3530.9	1.00
Cirrhosis (positive/negative)	67/42	28/19	0.82
Hepatitis (positive/negative)	96/13	42/5	0.82
Number of nodules (1/≥2)	87/22	33/14	0.35
Lesion diameter (Mean ± SD) (cm)	4.2 ± 2.9	3.9 ± 3.3	0.57
Treatment method (resection/ablation)	33/76	18/29	0.15
ER (%)	50/109(45.9)	25/47(53.2)	0.51
PT-E positive rate (%)	23/109 (21%)	16/47 (34%)	0.13
T-RO risk score (mean ± SD)	0.46 ± 0.28	0.43 ± 0.36	0.58
PT-RO risk score (mean ± SD)	0.46 ± 0.26	0.44 ± 0.29	0.67

*SD* standard deviation, *AFP* alpha-fetoprotein. Hepatitis, Hepatitis B or C; *ER* early recurrence, *PT-E* peritumoral enhancement, *T-RO* tumoral radiomics, *PT-RO* peritumoral radiomics, T-RO risk score refers to the application of T-RO model to the image of the cases in the training and validation sets, and obtain the risk score of each case (the output is the specific value, 0–1). PT-RO risk score refers to the application of PT-RO model to the image of the cases in the training and validation sets, and obtain the risk score of each case (the output is the specific value, 0–1). *P*-value reflected the differences between the training and validation cohorts, and P values of less than 0.05 (two-sided) were considered statistically significant

### Radiomics model-based prediction of early recurrence

Every patient had three DICOM images, including routine unenhanced images, hepatic arterial phase images and portal venous phase images; every image was used to extract two ROIs (T-RO and PT-RO). A total of 1044 extracted features were extracted from every ROI, and in all of the extracted features, approximately 43% were ICC ≥ 0.6. Then, based on the training cohort, this 43% of the 1044 features was reduced to 6 potential predictors both in the T-RO and PT-RO models using the LASSO regression model.

PT-RO model:

3.133089–39.22685*InverseDifferenceMoment_AllDirection_offset2_SD (routine unenhanced phase) + 1.004993 × 10^− 5^*ClusterShade_AllDirection_offset9_SD (routine unenhanced phase) + 1.827011 × 10^− 5^*ClusterShade_AllDirection_offset1_SD (hepatic arterial phase) - 2.826571*LongRunEmphasis_angle90_offset5 (hepatic arterial phase) - 6.908005 × 10^− 6^*ClusterShade_AllDirection_offset5 (portal venous phase) + 1.170673 × 10^− 2^*HighGreyLevelRunEmphasis_AllDirection_offset8_SD (portal venous phase)

T-RO model:

− 17.25829 **+** 2.491076 × 10^2^*AngularSecondMoment (routine unenhanced phase)

-10.46937*inverseDifferenceMoment (routine unenhanced phase) -3.584612 × 10^− 3^* Quantile0.025 (hepatic arterial phase)

-64.52834*InverseDifferenceMoment_AllDirection_offset2_SD (hepatic arterial phase) -7.422241 × 10^− 6^*ClusterShade_angle0_offset7 (portal venous phase) + 18.42472*ShortRunEmphasis_angle90_offset9 (portal venous phase).

### Evaluating overfitting of the prediction models between the training and validation sets

AUC values were measured to demonstrate overfitting of the PT-RO model, T-RO model and PT-E (Table 2). The PT-RO model yielded an AUC of 0.80 (95% CI, 0.72 to 0.89) in the training cohort and 0.79 (95% CI, 0.66 to 0.92) in the validation cohort with no significant difference between cohorts (*P* = 0.47). The T-RO model yielded an AUC of 0.82 (95% CI, 0.74 to 0.90) in the training cohort and 0.62 (95% CI, 0.46 to 0.79) in the validation cohort with a significant difference between cohorts (*P* < 0.01), which demonstrated extreme overfitting. The PT-E yielded an AUC of 0.64 (95% CI, 0.56 to 0.72) in the training cohort and 0.61 (95% CI, 0.47 to 0.74) in the validation cohort with no significant difference between cohorts (*P* = 0.11).

**Table 2:** Evaluating the overfitting of the prediction models

Models	AUC [95%CI]		*P*
	Training Set	Validation Set	
PT-RO	0.80 [0.72, 0.89]	0.79 [0.66, 0.92]	0.47
T-RO	0.82 [0.74, 0.90]	0.62 [0.46, 0.79]	< 0.01
PT-E	0.64 [0.56, 0.72]	0.61 [0.47, 0.74]	0.11

*AUC* area under the curve, *CI* Confidence Interval, *PT-RO* peritumoral radiomics, *T-RO* tumoral radiomics, *PT-E* peritumoral enhancement; *P*-value reflected the differences between the training and validation cohorts, and P values of less than 0.05 (two-sided) were considered statistically significant

### Evaluation and comparison of prediction performance in the validation set

#### Prediction accuracy

**Fig. 3:**
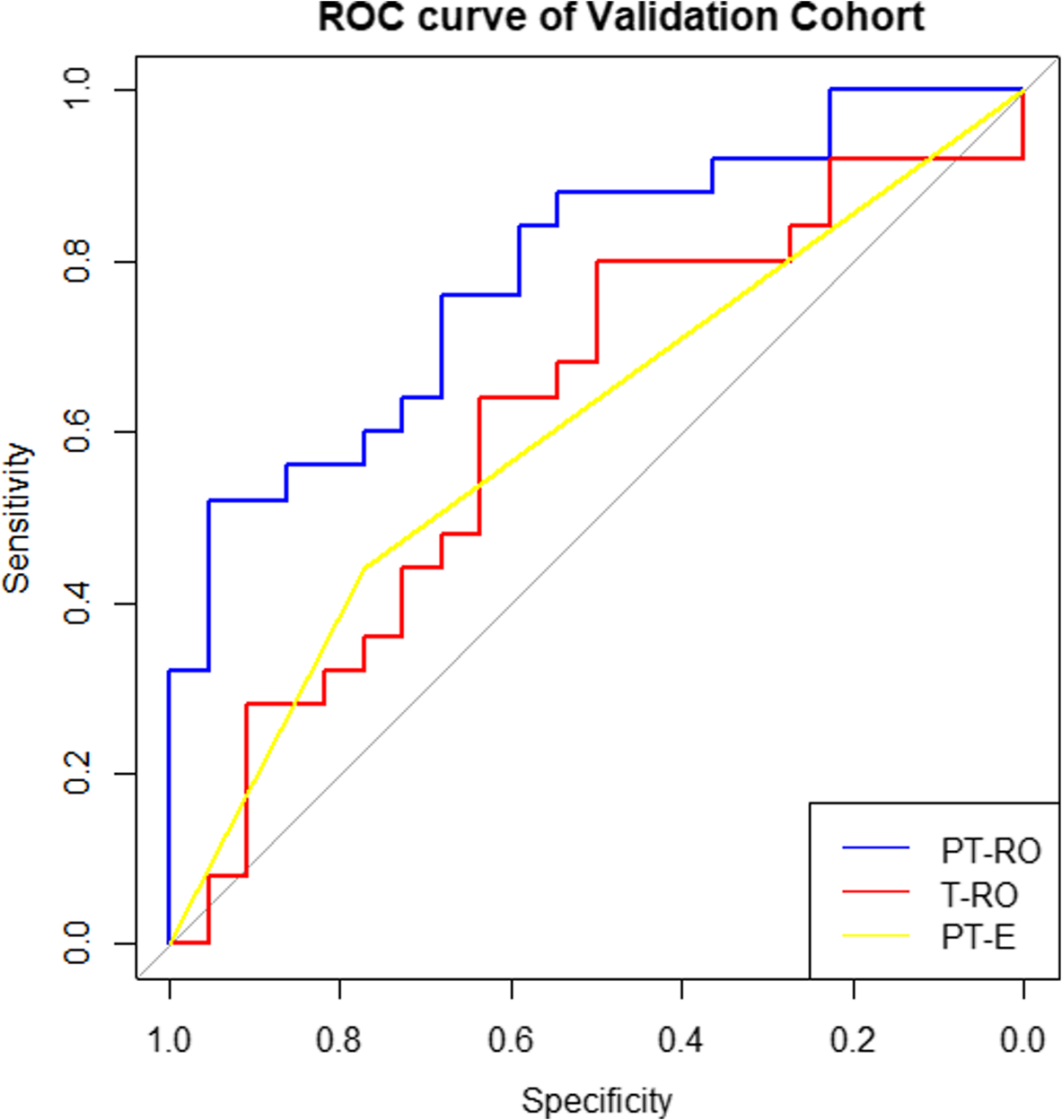
Receiver operating characteristic (ROC) curves of the PT-RO model (blue color), T-RO model (red color) and PT-E (yellow color) performed in the validation cohort

The ROC curves of the two radiomics models and PT-E were plotted to show the prediction accuracy in the validation cohort (Fig. 3). AUC values were measured to quantify the prediction accuracy of the radiomics models and PT-E (Table 3). The AUC of the PT-RO model was significantly higher than that of the T-RO model (*P* < 0.01) or PT-E (P < 0.01) in the validation cohort. The positive predictive value (PPV) of the PT-RO model was significantly higher than that of the T-RO model (P < 0.01) or PT-E (P < 0.01) in the validation cohort, while the negative predictive value (NPV) of the PT-RO model was similar with that of the T-RO model (*P* = 0.92) and PT-E (*P* = 0.38).

**Table 3:** Evaluating the performance of the prediction models

Models	AUC		cfNRI			IDI		PPV		NPV	
	AUC [95%CI]	P	cfNRI+	cfNRI-	P	IDI	P	PPV	P	NPV	P
PT-RO	0.79 [0.66, 0.92]	–	–	–	–	–	–	0.93	–	0.64	–
T-RO	0.62 [0.46, 0.79]	< 0.01	−0.47	−0.32	< 0.01	0.22	< 0.01	0.63	< 0.01	0.65	0.92
PT-E	0.61 [0.47, 0.74]	< 0.01	−0.24	− 0.41	0.02	0.20	0.01	0.69	< 0.01	0.55	0.38

*AUC* area under the curve, *CI* Confidence Interval, cfNRI+: movement in predicted risks introduced by changes of models in ER cases. cfNRI*-:* movement in predicted risks introduced by changes of models in non-ER cases. *IDI* Integrated Discrimination Improvement, *PPV* positive predictive value, *NPV* negative predictive value; *P* values of less than 0.05 (two-sided) were considered statistically significant; *PT-RO* peritumoral radiomics, *T-RO* tumoral radiomics, *PT-E* peritumoral enhancement

#### Calibration

**Fig. 4:**
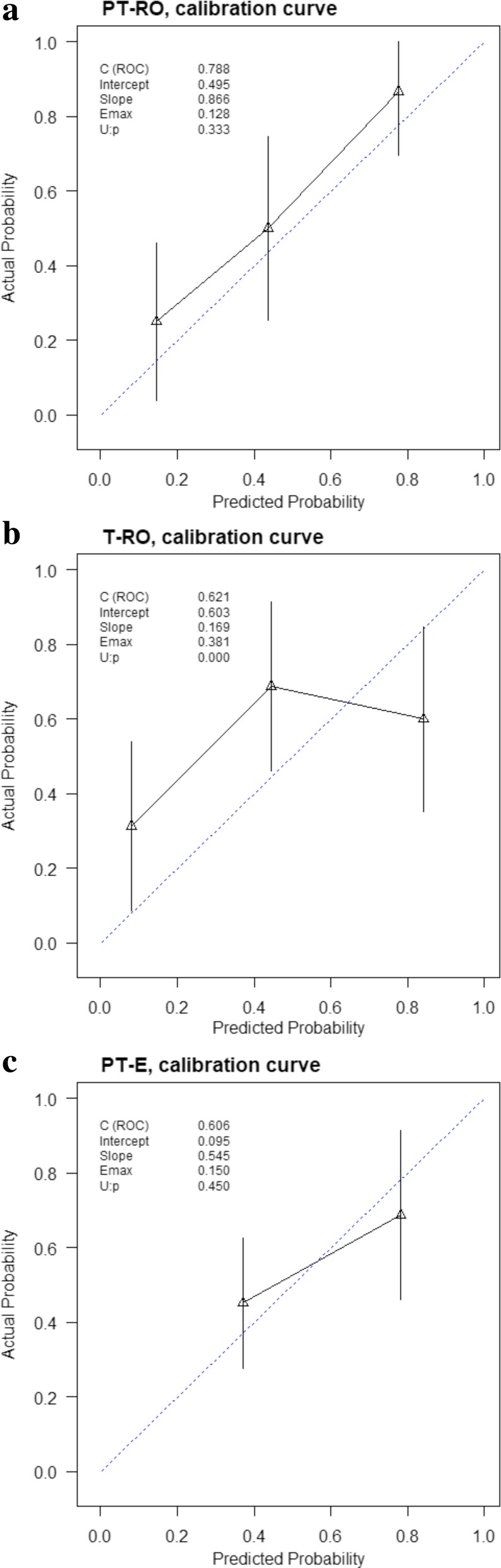
Calibration curves of the PT-RO model (**a**), T-RO model (**b**) and PT-E (**c**) performed in the validation cohort. The calibration curves depict the calibration of the models in terms of agreement between the predicted risks and the observed outcomes of HCC early recurrence. The solid line represents the performance of the models, and the dotted line represents an ideal model. The closer solid line is to the dotted line, the better the calibration

The calibration curves of the PT-RO model (Fig. 4a), T-RO model (Fig. 4b) and PT-E (Fig. 4c) applied to the validation cohort are shown. To evaluate whether the prediction models were well-calibrated, the unreliability (U) statistics were calculated to reflect the reliability of the calibration curves. The PT-RO model and PT-E showed reliable results for the calibration curves (*P* > 0.05), meaning that the PT-RO model and PT-E showed good agreement between prediction and observation. However, the T-RO model was not well-calibrated (*P* < 0.01), indicating poor agreement between prediction and observation.

#### Clinical application

**Fig. 5:**
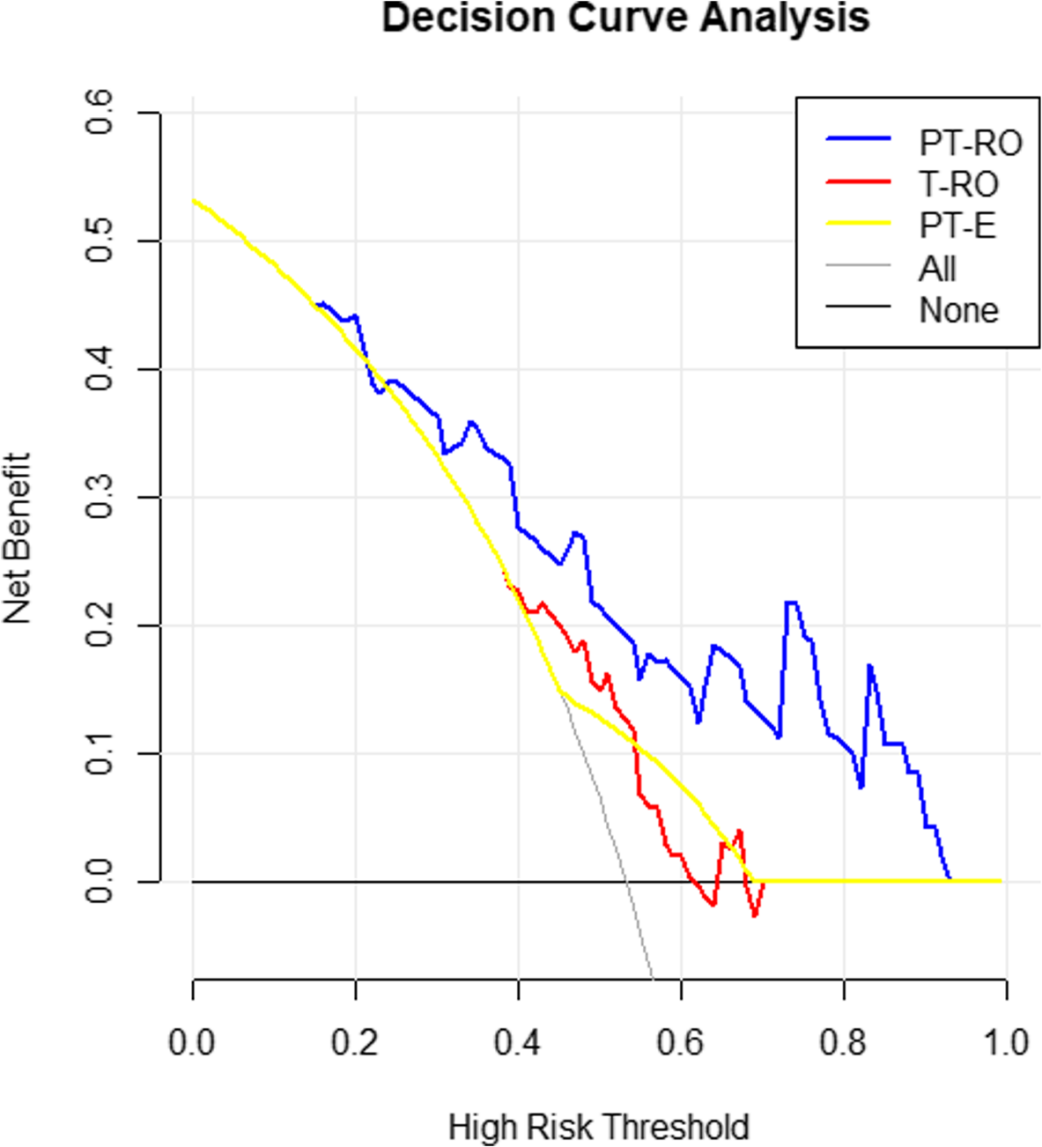
Decision curves of the PT-RO model (blue color), T-RO model (red color) and PT-E (yellow color) performed in the validation cohort

DCA for the two radiomics models and PT-E was performed in the validation cohort (Fig. 5). The highest curve (representing the PT-RO model) at any given threshold probability is the optimal decision-making strategy to maximize the net benefit compared with other models. Hence, the DCA showed that the PT-RO model had the highest overall net benefit compared with either the T-RO model or PT-E. CfNRI and IDI were measured to quantify the prediction accuracy of the radiomics models and PT-E (Table 3). CfNRI was used to evaluate if the radiomics models and PT-E addition led to a better reclassification of patients. The cfNRI indicated that the PT-RO model could correctly reclassify 47% of ER cases and 32% of non-ER cases compared to the T-RO model (P < 0.01), and the PT-RO model could correctly reclassify 24% of ER patients and 41% of non-ER cases compared to PT-E (*P* = 0.02). IDI indicated that the PT-RO model could improve prediction accuracy by 0.22 (P < 0.01) compared to the T-RO model and 0.20 (*P* = 0.01) compared to PT-E.

## Discussion

To the best of our knowledge, this is the first study to develop a PT-RO model to predict the ER of HCC. The performance of this model was validated in an independent validation cohort with respect to overfitting, prediction accuracy, calibration and clinical application. The PT-RO model was superior to the T-RO model and the conventional imaging feature PT-E.

PT-E can reflect hemodynamic perfusion changes of HCC with MVI [11], which is useful for predicting MVI of HCC and the risk of ER. Our study found that PT-E was a significant indicator of ER, which was similar to previous reports [13, 14, 16, 30–32]. However, the prediction accuracy was low; in our study, the AUC of PT-E was only 0.61 (95% CI, 0.47 to 0.74). Compared with conventional CT imaging features, radiomics features capture more information objectively and quantitatively at low cost and may help predict clinical outcomes [17].

In our study, AK software was used to extract the imaging features of peritumoral and intratumoral regions. A total of 1044 features were extracted from every ROI, and approximately 43% of the extracted features were ICC ≥ 0.6. Then, based on the training cohort, this 43% of the 1044 features was reduced to 6 potential predictors using the LASSO regression model to build both the PT-RO and the T-RO radiomics prediction models. The PT-RO model demonstrated outstanding discrimination in both the training (AUC, 0.80; 95% CI, 0.72 to 0.89) and validation (AUC, 0.79; 95% CI, 0.66 to 0.92) cohorts. The high AUC suggested that the PT-RO model performed well in discriminating for ER. In addition, the PT-RO model was compared with the T-RO model and PT-E. In our study, the PT-RO model significantly improved the accuracy of the preoperative model for predicting ER compared with the T-RO model and PT-E (both *P* < 0.01). Moreover, compared with the T-RO model and PT-E, the PT-RO model had a similar NPV, but the PPV was significantly higher, which provided a reference to make a closer follow-up plan for patients who were predicted to be positive for ER.

Prior studies have reported CT-based radiomics models for predicting the prognosis of HCC [21, 22]. Cozzi, et al. [21] developed a CT-based radiomics prediction model that showed an accuracy of 80.0% in predicting overall survival in HCC patients (with a maximum follow-up of 28 months). Zhou, et al. [22] developed a CT-based radiomics model that demonstrated an AUC of 0.82 in predicting the early recurrence (≤1 year) of HCC. These two available studies were all based on T-RO models. However, these two studies lacked validation based on independent datasets, which may lead to a risk of overfitting the analyses [18]. In our study, the T-RO model demonstrated significant overfitting (AUC of 0.82 in the training cohort and 0.62 in the validation cohort, *P* < 0.01). This overfitting may be associated with the great heterogeneity of the whole tumor [33].

Our study used a peritumoral ROI delineated with a 2 cm expansion from the lesion, which was based on the current standard for resectioning margins for HCC. A randomized controlled trial reported that a margin aiming at 2 cm could decrease the postoperative recurrence rate and improve survival outcomes [34]. Radiomics features extracted from a 2 cm peritumoral ROI were most likely to provide important information for predicting ER.

The calibration curve of the predictive model demonstrates good agreement between the predictive and actual probabilities when the *P* value is more than 0.05. In our study, the calibration curve showed that the predicted effect of the PT-RO model had better agreement with the actual HCC recurrence situation in the validation cohort than that of the T-RO models (U: *P* = 0.33 vs. U: P < 0.01). The calibration curve also showed that the predicted effect of the PT-E model was in good agreement with the actual HCC recurrence situation the validation cohort (U: *P* = 0.45). Notably, DCA showed that the PT-RO model adds more benefit to predicting ER than the T-RO model and PT-E at any given threshold probability.

Our study had several limitations. First, this was a retrospective single-center study. Inevitably, some bias may exist or have affected the analysis. Second, we used internal validity rather than external validity, making it difficult to generalize the outcomes to other institutions. And our results of an Asian population may not be generalizable for a Western population. Third, radiomics features were extracted from the largest cross-sectional area instead of the whole tumor, which may provide more information. In our current study, the software we used did not have the 3D feature extraction function at the time of analysis. At present, the features based on a single slice have shown a strong correlation with prognosis. In addition, 2D features are easier to obtain, are less labor intensive, have lower complexity and allow for faster calculations. Fourth, the local recurrence rate after tumor ablation is higher compared to the local recurrence rate after tumor resection, which might therefore cause a potential bias. As the small sample size in our study makes it difficult to perform subgroup analyses between patients undergoing tumor ablation and tumor resection, larger studies should be performed to enable subgroup analyses. Therefore, although this study provided initial evidence that the PT-RO model can be useful for predicting the ER of HCC, more prospective studies should be performed to validate our results.

## Conclusion

In conclusion, the present study indicates that a PT-RO signature is a powerful preoperative predictor for the ER of HCC and that the PT-RO model is better than the T-RO model and PT-E. Such quantitative radiomics prognostic models of HCC may potentially be useful for precision medicine and affect patient treatment strategies.

## Additional file

Additional file 1: Detailed introduction of calibration curves and decision curves, category-free Net Reclassification Index and integrated discrimination improvement. (DOCX 20 kb)

## Abbreviations

AFP: Alpha-fetoprotein
AUC: Area under the curve
CECT: Contrast-enhanced computed tomography
CEMR: Contrast-enhanced magnetic resonance imaging
CEUS: Contrast-enhanced ultrasound
CfNRI: Category-free Net Reclassification Index
CT: Computed tomography
DCA: Decision curve analysis
DICOM: Digital imaging data and communications in medicine
ER: Early recurrence
HCC: Hepatocellular carcinoma
ICC: Inter-class correlation coefficients
IDI: Integrated Discrimination Improvement
LASSO: Least Absolute Shrinkage and Selection Operator
MVI: Microvascular invasion
NPV: Negative predictive value
PPV: Positive predictive value
PT-E: Peritumoral enhancement
PT-H: Peritumoral hypointensity on hepatobiliary phase
PT-RO: Peritumoral radiomics
ROC: Receiver Operating Characteristic
ROI: Region of interest
TACE: Transarterial chemoembolization
T-RO: Tumoral radiomics
