# Supplementary material for: CT-based peritumoral radiomics signatures to predict early recurrence in hepatocellular carcinoma after curative tumor resection or ablation
Source: Cancer Imaging. 2019 Feb 27;19:11. doi: 10.1186/s40644-019-0197-5 (PMC6391838; doi:10.1186/s40644-019-0197-5)
Supplement: Supplementary file 1 — Detailed introduction of calibration curves and decision curves, category-free Net Reclassification Index and integrated discrimination improvement. (DOCX 20 kb) [file 40644_2019_197_MOESM1_ESM.docx]

Supplementary Material

Thorough evaluation of a predictive model is important to validate its ability for use in decision-making or broader simulation modeling. Measures of discrimination, such as sensitivity, specificity and receiver operating characteristics, are commonly used to evaluate how well the model can distinguish between the possible outcomes. However, these discrimination tests cannot confirm that the predicted probabilities are accurate and without bias. Calibration curves typically measure the accuracy of predicted probabilities by comparing them to mean event occurrence rates within groups of similar test records [1]. The guidelines for the selection of an appropriate regression model by calibration curves have been defined in regulatory guidance documents and lead publications by subject matter experts [2].

Decision curve analysis is a novel method for evaluating prediction models, which was developed in 2006 by Dr. Andrew Vickers from Memorial Sloan-Kettering Cancer Center [3]. Decision curve analysis is a method for evaluating and comparing prediction models that incorporates clinical consequences, requires only the data set on which the models are tested, and can be applied to models that have either continuous or dichotomous results. Decision curve analysis identifies the range of threshold probabilities in which a model is of value, the magnitude of benefit, and which of the models are optimal. The key concept for this type of evaluation is the "net benefit", and decision curve analysis plots net benefit at a range of clinically reasonable risk thresholds [4; 5]. A decision curve shows the performance of a risk model in a population in which every patient has the same expected benefit and cost of intervention, and is an improvement over purely mathematical measures of performance such as the area under the receiver operating characteristic curve [6].

Category-free Net Reclassification Index [7] and integrated discrimination improvement values [8] refer to the improved classification of patients using the reference model and the new model. The net reclassification index, as originally proposed, seeks to quantify whether a new marker provides clinically relevant improvements in prediction. In the definition of “net reclassification indices,” the risk prediction model with established predictors is called the “old” model. The model that adds the new marker is the “new” model. “Events” are cases which comprises persons who have or will have the disease or the outcome in the absence of intervention. “Nonevents” are controls. The formula defining the net reclassification index (NRI) is [9]:

NRI = P(up|event)−P(down|event) + P(down|nonevent)−P(up|nonevent) (1)

where “up” means that the new risk model places a person into a higher risk category than the old model. Similarly, “down” means the new model places a person into a lower risk category.

The definition of the net reclassification index in Equation (1), based originally on discrete predefined risk categories, generalizes to any upward or downward movement in predicted risks [10]. The “category-free net reclassification index” (also called “continuous net reclassification index”) is a new descriptive device designed to avoid predefined risk categories and interpreted definitions (1) this way [7].

Integrated discrimination improvement values are commonly used to compare two risk prediction models; the values summarize the extent to which a new model increases risk in events and decreases risk in nonevents [8].

Let p_newi_ denote the risk of the event of interest for an individual i under the new model, and p_oldi_ denote the risk of the event of interest for an individual i under the old model, where p̄_new_ is the average new model risk prediction for the n individuals, and p̄_old_ is the average old model risk predictions for the n individuals. In addition, we introduce a subscript indicating whether an individual i has the event of interest (1 if they have the event of interest, and 0 otherwise). The integrated discrimination improvement value represents the extent to which the new model increases the average risk in events and decreases the average risk in nonevents, which can be written as:

Integrated discrimination improvement value = (p̄_new.1_ − p̄_old.1_) − (p̄_new.0_ − p̄_old.0_) [8]

**References:**

1. Fenlon C, O'Grady L, Doherty ML, Dunnion J. A discussion of calibration techniques for evaluating binary and categorical predictive models. Prev Vet Med. 2018;149:107-14.
2. Azadeh M, Gorovits B, Kamerud J, et. al. Calibration Curves in Quantitative Ligand Binding Assays: Recommendations and Best Practices for Preparation, Design, and Editing of Calibration Curves. AAPS J. 2017;20:22.
3. Vickers AJ, Elkin EB. Decision curve analysis: a novel method for evaluating prediction models. Med Decis Making. 2006;26:565-74.
4. Rousson V, Zumbrunn T. Decision curve analysis revisited: overall net benefit, relationships to ROC curve analysis, and application to case-control studies. BMC Med Inform Decis Mak. 2011;11:45.
5. Van Calster B, Wynants L, Verbeek JFM, et. al. Reporting and Interpreting Decision Curve Analysis: A Guide for Investigators. Eur Urol. 2018;74:796-804.
6. Kerr KF, Brown MD, Zhu K, Janes H. Assessing the Clinical Impact of Risk Prediction Models With Decision Curves: Guidance for Correct Interpretation and Appropriate Use. J Clin Oncol. 2016;34:2534-40.
7. Kerr KF1, Wang Z, Janes H, et. al. Net reclassification indices for evaluating risk prediction instruments: a critical review. Epidemiology. 2014;25:114-21.
8. Chipman J, Braun D. Simpson's paradox in the integrated discrimination improvement. Stat Med. 2017;36:4468-81.
9. Pencina MJ, D'Agostino RB Sr, D'Agostino RB Jr, Vasan RS. Evaluating the added predictive ability of a new marker: from area under the ROC curve to reclassification and beyond. Stat Med. 2008; 27:157-72; discussion 207-12.
10. Pencina MJ, D'Agostino RB Sr, Steyerberg EW. Extensions of net reclassification improvement calculations to measure usefulness of new biomarkers. Stat Med. 2011; 30:11-21.
